# Impact of a three-subject, dual-track, interactive care model on hospitalized patients with diabetic foot

**DOI:** 10.3389/fendo.2026.1956826

**Published:** 2026-09-21

**Authors:** Yunxia Shi, Xiaoxiao Wang, Kewei Zhang, Xiaoning Shi, Bing Qin, Jie Kou

**Affiliations:** 1 Department of Vascular Surgery, Henan Provincial People’s Hospital, Zhengzhou, Henan, China; 2 Department of Psychiatry, Henan Provincial People’s Hospital, Zhengzhou, Henan, China; 3 Department of Nursing, Henan Provincial People’s Hospital, Zhengzhou, Henan, China

**Keywords:** diabetic foot, psychological status, quality of life, three-subject dual-track interactive care, wound healing

## Abstract

**Background:**

Diabetic foot (DF) is a severe chronic complication of diabetes, associated with high amputation risk and mortality. Conventional nursing often neglects patients’ psychological needs and long-term self-management, limiting comprehensive rehabilitation. Interactive nursing has shown promise in chronic disease care, yet systematic evaluations of its multidimensional effects remain scarce. This study assessed a three-subject, dual-track, interactive care (TSDTIC) model on wound healing, psychological state, and quality of life in DF patients.

**Methods:**

This retrospective cohort study included 150 hospitalized DF patients (June 2021 – June 2024). Patients were assigned to groups based on the nursing care model implemented during their admission period: the RC group (n=84) comprised patients admitted between June 2021 and December 2022, and the TSDTIC group (n=66) comprised patients admitted between January 2023 and June 2024. The TSDTIC model was introduced as a structured quality improvement program in January 2023; therefore, group assignment was sequential rather than randomized. Baseline demographic and clinical characteristics were comparable between groups (all P > 0.05), and ANCOVA with baseline values as covariates was performed for continuous outcomes. Outcomes included wound improvement rate (based on area reduction), fasting and postprandial glucose, Hamilton Anxiety/Depression Scale (HAMA/HAMD) scores, SF-36 quality-of-life scores, and nursing satisfaction.

**Results:**

The TSDTIC group showed significantly higher wound improvement rate (95.45% vs. 82.14%, P = 0.013). Post-intervention, fasting (6.29 ± 1.05 vs. 7.94 ± 1.82 mmol/L, P<0.001) and postprandial glucose (8.92 ± 1.29 vs. 10.35 ± 1.41 mmol/L, P<0.001) were markedly lower in TSDTIC. HAMA (7.21 ± 2.13 vs. 10.02 ± 2.06) and HAMD (6.88 ± 2.06 vs. 10.26 ± 2.14) scores significantly improved (both P<0.001). All eight SF-36 dimensions were higher in TSDTIC (all P<0.001 after Bonferroni correction). Satisfaction rate was higher in TSDTIC (95.45% vs. 73.81%, P<0.001).

**Conclusion:**

The TSDTIC model was associated with improvements in short-term wound healing, glycemic control, mental health, and quality of life in DF patients. However, given the historical control design and lack of long-term outcomes, findings should be interpreted cautiously; prospective randomized trials are warranted.

## 1 Introduction

Diabetic foot (DF) refers to a spectrum of pathological conditions affecting the foot in patients with diabetes, encompassing neuropathy, peripheral arterial disease, infection, and ulceration, which may progress to deep tissue destruction and lower extremity amputation (1–3). Approximately 537 million adults worldwide have diabetes, of which 19%–34% will develop DF ulcers in their lifetime; the lifetime incidence of lower limb amputation is 20%, and the five-year mortality rate is as high as 50%–70% (4). These estimates represent pooled or global data and may vary by region and population. As the prevalence of diabetes continues to increase worldwide, DF has become an important clinical and public health problem (5, 6). The healing process of foot ulcers in patients with diabetes is influenced by multiple factors, including blood glucose control, severity of local infection, health of the lower limb vessels, nerve function, and overall nutritional status (7–10). In recent years, although treatment methods, such as wound debridement, negative pressure drainage, and vascular reconstruction, have continued to improve, there remains a considerable number of patients with slow-to-heal wounds, repeated infection(s), and some who encounter the risk for amputation (11–13).

Conventional nursing models often prioritize hospital-based wound treatment and pharmacotherapy, with less systematic emphasis on patients’ psychological state, self-management ability, and continuous nursing after discharge. Studies have shown that negative emotions, such as anxiety and depression, are common in patients with DF (14, 15). In addition, DF has a long treatment cycle, and patients often encounter problems such as lack of nursing knowledge, poor compliance, and nonstandard follow-up after discharge, which leads to a high recurrence rate (16, 17). As a new, patient-centered nursing concept emphasizing the collaborative participation of nurses and patients, interactive nursing has demonstrated unique advantages in chronic disease management in recent years (18, 19). The three-subject, dual-track model, which integrates in-hospital specialized care with out-of-hospital continuous support, and coordinates the efforts of medical staff, patients, and family/community, represents a systematic approach to bridging the gaps in current DF care.

Wound healing is directly influenced by glycemic control, nutritional status, and infection management; psychological distress (anxiety/depression) impairs self-management and immune function via hypothalamic-pituitary-adrenal axis activation; enhanced self-management and social support improve glycemic control and treatment adherence; and these factors collectively determine health-related quality of life. The TSDTIC model targets these interdependent pathways through multidisciplinary in-hospital care and continuous out-of-hospital support. Furthermore, the TSDTIC model incorporates specific components designed to improve glycemic control, including structured diabetes education (dietary guidance, medication adherence counseling), one-on-one blood glucose self-monitoring training, establishment of individualized glycemic targets (fasting <7.0 mmol/L, 2h postprandial <10.0 mmol/L), and continuous feedback through WeChat groups and community nurse follow-up. These components are intended to empower patients to achieve better acute glycemic self-management, which is expected to improve fasting and postprandial glucose levels during hospitalization. Anxiety and depression are common among DF patients due to the risk of amputation, prolonged treatment burden, and functional limitations. Negative emotions can activate the hypothalamic-pituitary-adrenal axis and elevate cortisol levels, further impairing immune function and wound healing. Interactive nursing addresses these psychological factors through systematic screening, professional counseling, and enhanced social support networks, thereby potentially improving self-efficacy and treatment compliance.

Although multidisciplinary teams (MDTs) and transitional care programs have been reported, most of the existing models focus on in-hospital multidisciplinary coordination or post-discharge transitional care, but rarely integrate the two in an interactive framework (20, 21). TSDTIC is unique in that it involves health care providers, patients, and family/community caregivers simultaneously, spanning two continuous tracks inside and outside the hospital, with a structured interaction mechanism rather than a sequential handover. This creates a closed-loop system in which in-hospital education and empowerment translate directly into self-management and community support after discharge. Therefore, this retrospective cohort study was designed to evaluate the effects of this integrated model on wound healing, psychological status, and quality of life in patients with DF, with the aim of providing practice-based evidence for optimizing DF nursing programs.

## 2 Methods

### 2.1 Patients

This was a retrospective cohort study with a historical control design. Patients were divided into two groups according to the nursing care model implemented during their admission period: the RC group (n=84) included patients admitted between June 2021 and December 2022; the TSDTIC group (n=66) included patients admitted between January 2023 and June 2024. The TSDTIC model was introduced as a structured quality improvement program in January 2023; therefore, group allocation was sequential rather than randomized. The different sample sizes reflect the number of patients with different recruitment durations. No patient withdrew after enrollment, and no patient died in the hospital. The inclusion criteria were as follows: age ≥ 18 years; fulfilling the World Health Organization diagnostic criteria for diabetes; fulfilling the diagnostic criteria for DF (Wagner grade 1–4); wound area > 2 cm²; presence of clear consciousness and basic communication skills; and complete clinical data. The exclusion criteria were as follows: severe heart, liver, or kidney dysfunction, or malignant tumor; Wagner grade 5 (full-foot gangrene) or amputation; other causes of lower extremity ulcers (such as venous ulcers, arterial ulcers, or pressure injuries); history of mental illness or cognitive impairment; pregnant or lactating women; and incomplete data or loss to follow-up. The study protocol was reviewed and approved by the hospital’s institutional review board (IRB), which waived the requirement for informed consent because the analysis used retrospectively collected, anonymized data from a quality improvement initiative that represented standard care practices; all interventions were within the scope of routine clinical nursing, and no experimental procedures were involved.

### 2.2 Care methods

RC group: This group mainly received health education upon admission, blood glucose monitoring, dressing changes for wounds as per attending physician’s instructions, routine guidance upon discharge, and follow-up visits at the outpatient department. In terms of psychological care, only routine comforting communication was offered, with no systematic psychological assessment or intervention.

The TSDTIC group was constructed under the theoretical guidance of the dual-track parallel and three-subject collaboration. The three subjects participating in the TSDTIC model are explicitly defined as: (1) Medical staff, including wound specialist nurses, attending physicians, psychotherapists, and dietitians, responsible for clinical assessment, wound management, psychological screening, and nutritional intervention; (2) Patients, serving as active participants in self-monitoring, self-care, and shared decision-making; and (3) Family/community caregivers, including family members providing daily support and community nurses conducting home visits and telephone follow-up. These three subjects interact through structured communication channels (multidisciplinary rounds, WeChat groups, and community nursing networks) to ensure coordinated care across both tracks. The implementation process was divided into the following 2 components.

In-hospital nursing: The entire patient process from admission to discharge was implemented with multidisciplinary collaborative nursing led by wound specialist nurses. Specific contents include conducting standardized wound assessments on a weekly basis; implementing personalized debridement and dressing selection; performing precise management of blood sugar, with explicit glycemic targets of fasting blood glucose < 7.0 mmol/L and 2-hour postprandial blood glucose < 10.0 mmol/L, dynamically adjusted according to individual patient conditions; conducting nutritional risk assessment and intervention; and providing systematic psychological screening and counseling.

Out-of-hospital care: This focuses on the continuation of care after discharge, establishing a link among hospitals, communities, and families. Specific measures included designing a personalized home-care plan 2 days before discharge, establishing a WeChat group of patients, and specialist nurses responding to questions online twice per week. The WeChat group adherence was monitored through message read receipts and active participation records; the active engagement rate was 81.8% (54/66 patients), defined as patients who read health education messages and responded to nurse-initiated inquiries at least once per week. Additionally, the quality of engagement was qualitatively monitored by nurses’ documentation of patient-raised questions and the clarity of responses, though no formal scoring system was applied. Community nurses were arranged to follow up once every 2 weeks in the first 3 months, and then monthly telephone follow-up thereafter. The scheduled follow-up completion rate by community nurses was 87.9% (58/66 patients), with missed follow-ups primarily due to patient relocation (n=4), hospitalization for other conditions (n=2), and patient refusal (n=2). Simultaneously, a green follow-up channel was set up to prioritize outpatient wound treatment for patients.

The primary outcomes (wound healing, glucose, psychological status, quality of life) were assessed at discharge and reflect the in-hospital component of TSDTIC. The out-of-hospital component (WeChat support, community follow-up) was designed to ensure continuity of care and prevent recurrence after discharge, but its direct effects on these specific short-term outcomes were not measured in this study. The inclusion of post-discharge care in the intervention description reflects the complete model design, while the reported outcomes represent short-term in-hospital effects.

### 2.3 Observation indicators

Effective rate of wound improvement: Wound healing was evaluated quantitatively based on the percentage reduction in wound area from admission to discharge. The criteria were defined as: markedly effective (wound area reduction ≥80% and no signs of infection), effective (wound area reduction ≥40% but <80%, with reduced exudate and granulation tissue formation), and ineffective (wound area reduction <40% or worsened/developed new infection). These thresholds were investigator-defined based on clinical practice and the need for categorical analysis of wound area reduction during hospitalization; they are not derived from an established international guideline. The ≥80% threshold represents near-complete healing, 40–80% represents substantial improvement with granulation tissue formation, and <40% represents minimal change or deterioration. The evaluation was performed by two independent wound care nurses who were blinded to the group assignment; any disagreement was resolved by consensus. The effective rate of wound improvement was calculated as (number of markedly effective + effective cases)/total cases ×100. Wound area was measured using digital photography followed by planimetry analysis with ImageJ software (National Institutes of Health, USA). Photographs were taken after standardized wound cleaning and debridement by wound care nurses. A standardized ruler was placed adjacent to the wound for calibration. Irregular wounds were measured by manual tracing of the wound margin on the digital image. The same procedure was applied at admission and discharge by two independent wound care nurses blinded to group assignment.

Blood glucose levels: Fasting blood glucose and 2 h postprandial blood glucose levels were measured before and after nursing. Glycated hemoglobin (HbA1c) was not included because this retrospective study focused on short-term glycemic control during hospitalization, and HbA1c data were not consistently available at the predefined assessment time points for all patients. Fasting and 2-hour postprandial blood glucose were selected as they directly reflect acute glycemic management and are routinely monitored in clinical practice.

Psychological status: The Hamilton Anxiety Scale (HAMA) and Hamilton Depression Scale (HAMD) were used to evaluate anxiety and depression of the 2 patient groups before and after nursing. The higher the scale score, the more obvious the negative emotions (22).

Quality of life: The Short-Form 36 (SF-36) scale was used as an evaluation tool, which included 8 dimensions: physical functioning, role-physical, bodily pain, vitality, social functioning, role-emotional, mental health, and general health, and a total of 36 items. The evaluation scores for each dimension range from 0 to 100 points (23).

Satisfaction with nursing: Patients were interviewed using a self-designed nursing satisfaction questionnaire at the authors’ hospital, scored from 0–100 points as follows: 85–100 (very satisfied); 60–84 (relatively satisfied); and 0–60 (dissatisfied). This questionnaire has not undergone formal reliability and validity testing; therefore, the satisfaction results should be interpreted as exploratory.

### 2.4 Statistical analysis

Data were plotted and analyzed using Prism version 8.0 (GraphPad Inc., San Diego, CA, USA). Measurement data are expressed as mean ± standard deviation (SD). An independent sample t-test was used for comparisons between the two groups, and a paired sample t-test was used for comparisons before and after nursing. For categorical variables, the chi-square test was used; when expected cell frequencies were <5, Fisher’s exact test was applied. For multiple comparisons of the 8 SF-36 dimensions, we used Bonferroni correction to control the family-wise error rate, with a corrected significance level of α’ = 0.05/8 = 0.00625; all P values for SF-36 were <0.001, thus remaining statistically significant. Additionally, to adjust for potential baseline differences in continuous outcomes (blood glucose and psychological scores), we performed analysis of covariance (ANCOVA) with baseline values as covariates; the results consistently showed significant group differences (all P<0.001). Differences with P < 0.05 were considered to be statistically significant.

## 3 Results

### 3.1 Baseline characteristics

Data from 150 patients with DF were divided into 2 groups: RC (*n* = 84 [48 male, 36 female; mean (± SD) age, 68.58 ± 5.51 years]); and TSDTIC (*n* = 66 [36 male, 30 female; mean age, 67.97 ± 6.20 years). There were no significant differences in age, sex, body mass index, disease course, wound site, education level, history of hypertension, smoking history, or alcohol consumption history between the 2 groups (*P* > 0.05). Additionally, no significant differences were observed in diabetes type, Wagner grade, baseline wound area, presence of infection at admission, peripheral vascular disease status, neuropathy status, nutritional status (NRS-2002), baseline HbA1c and duration of hospitalization between the two groups (all *P* > 0.05) (**Table 1**). No major changes in DF management protocols, antibiotic policies, wound care staffing, or hospital policies occurred between the two periods, based on hospital administrative records. The same senior attending physicians and wound care teams were involved throughout the study period.

**Table 1:** Comparison of general information of the two groups of patients.

Classification	RC group (n=84)	TSDTIC group (n=66)	t/χ2	P value
Gender, n (%)			0.101	0.750
Male	48 (57.14)	36 (54.55)		
Female	36 (42.86)	30 (45.45)		
Age, years	68.58 ± 5.51	67.97 ± 6.20	0.641	0.523
BMI, Kg/m²	23.81 ± 2.12	23.38 ± 2.20	0.867	0.388
Course of disease (week)	7.79 ± 2.37	7.58 ± 2.71	0.505	0.614
Diabetes type, n (%)			0.010	0.919
Type 1	6 (7.14)	5 (7.58)		
Type 2	78 (92.86)	61 (92.42)		
Wagner grade, n (%)			0.158	0.984
Grade 1	20 (23.81)	16 (24.24)		
Grade 2	28 (33.33)	23 (34.85)		
Grade 3	24 (28.57)	19 (28.79)		
Grade 4	12 (14.29)	8 (12.12)		
Baseline wound area, cm²	5.81 ± 3.22	6.13 ± 3.54	0.541	0.589
Infection at admission, n (%)	56 (66.67)	41 (62.12)	0.334	0.563
Peripheral vascular disease, n (%)	42 (50.00)	35 (53.03)	0.136	0.712
Neuropathy, n (%)	72 (85.71)	55 (83.33)	0.161	0.688
Nutritional status (NRS-2002), n (%)			0.105	0.747
Normal risk	34 (40.48)	25 (37.88)		
At risk	50 (59.52)	41 (62.12)		
Site of wound, n (%)			0.114	0.990
Heel	45 (53.57)	37 (56.06)		
Toe	16 (19.05)	12 (18.18)		
Ankle	13 (15.48)	10 (15.15)		
Other	10 (11.90)	7 (10.61)		
Educational level, n (%)			0.106	0.948
Primary school	47 (55.95)	36 (54.55)		
Secondary and high school	26 (30.95)	22 (33.33)		
College, Bachelor’s Degree and above	11 (13.10)	8 (12.12)		
History of hypertension, n (%)	30 (35.71)	22 (33.33)	0.093	0.761
Smoking history, n (%)	38 (45.24)	28 (42.42)	0.119	0.730
Drinking history, n (%)	24 (28.57)	20 (30.30)	0.053	0.817
Baseline HbA1c, %	8.53 ± 1.81	8.42 ± 1.93	0.327	0.744
Duration of hospitalization, days	18.5 ± 6.2	19.2 ± 5.8	0.712	0.478

RC, routine care; TSDTIC, three-subject dual-track interactive care; BMI, body mass index; NRS-2002, Nutritional Risk Screening 2002; HbA1c, glycated hemoglobin.

### 3.2 Effect on wound improvement

In the RC group, 69 cases achieved effective wound management, of which 41 were classified remarkable and 28 were effective. In the TSDTIC group, 63 cases achieved effective wound management, including 50 that were classified remarkable and 13 that were effective. Wound improvement was significantly greater in the TSDTIC group than in the RC group (*P* = 0.013) (**Table 2**).

**Table 2:** The effective rate of wound improvement was compared between the two groups.

Classification	RC group (n=84)	TSDTIC group (n=66)	χ2	P value
Markedly effective	41 (48.81)	50 (75.76)		
Effective	28 (33.33)	13 (19.70)		
No effect	15 (17.86)	3 (4.54)		
Total effective rate	69 (82.14)	63 (95.45)	6.202	0.013

RC, routine care; TSDTIC, three-subject dual-track interactive care. Wound healing was defined based on percentage area reduction, markedly effective (≥80% reduction), effective (≥40% to <80%), ineffective (<40%).

### 3.3 Blood glucose indicators

Before nursing, there were no statistically significant differences in 2 h postprandial blood glucose levels (13.64 ± 1.88 mmol/L *versus* [*vs*.] 13.56 ± 1.92 mmol/L; *P* = 0.792) and fasting blood glucose levels (9.39 ± 1.47 mmol/L vs. 9.24 ± 1.43 mmol/L; *P* = 0.529) between the RC and TSDTIC groups. After nursing, 2 h postprandial blood glucose and fasting blood glucose levels in the 2 groups decreased. In TSDTIC group, 2 h postprandial blood glucose (8.92 ± 1.29 mmol/L *vs*. 10.35 ± 1.41 mmol/L; *P* < 0.001) and fasting blood glucose (6.29 ± 1.05 mmol/L *vs*. 7.94 ± 1.82 mmol/L; *P* < 0.001) were significantly lower than those in the RC group (**Table 3**). ANCOVA adjusting for baseline glucose levels confirmed the significant effect of the intervention (both P<0.001).

**Table 3:** Compare the blood sugar levels of the two groups [(mean ± SD)].

Classification	2h postprandial blood glucose (mmol/L)		Fasting blood glucose (mmol/L)	
	Before nursing	After nursing	Before nursing	After nursing
RC group (n=84)	13.64 ± 1.88	10.35 ± 1.41	9.39 ± 1.47	7.94 ± 1.82
TSDTIC group (n=66)	13.56 ± 1.92	8.92 ± 1.29	9.24 ± 1.43	6.29 ± 1.05
t	0.264	6.354	0.632	6.569
P value	0.792	<0.001	0.529	<0.001

RC, routine care; TSDTIC, three-subject dual-track interactive care. ANCOVA with baseline values as covariates also confirmed significant group differences (both P<0.001).

### 3.4 Psychological status

Before nursing, there was no significant difference in HAMA (16.92 ± 2.10 *vs*. 16.85 ± 2.26) and HAMD (17.14 ± 2.04 *vs.* 17.02 ± 2.21) scores between the 2 groups (*P* > 0.05). After nursing, HAMA and HAMD scores decreased in both groups; however, HAMA (7.21 ± 2.13 *vs*. 10.02 ± 2.06) and HAMD (6.88 ± 2.06 *vs.* 10.26 ± 2.14) scores in the TSDTIC group were significantly lower than those in the RC group (*P* < 0.001) (**Figure 1**). ANCOVA with baseline scores as covariates also showed significant group differences (P<0.001).

**Figure 1:**
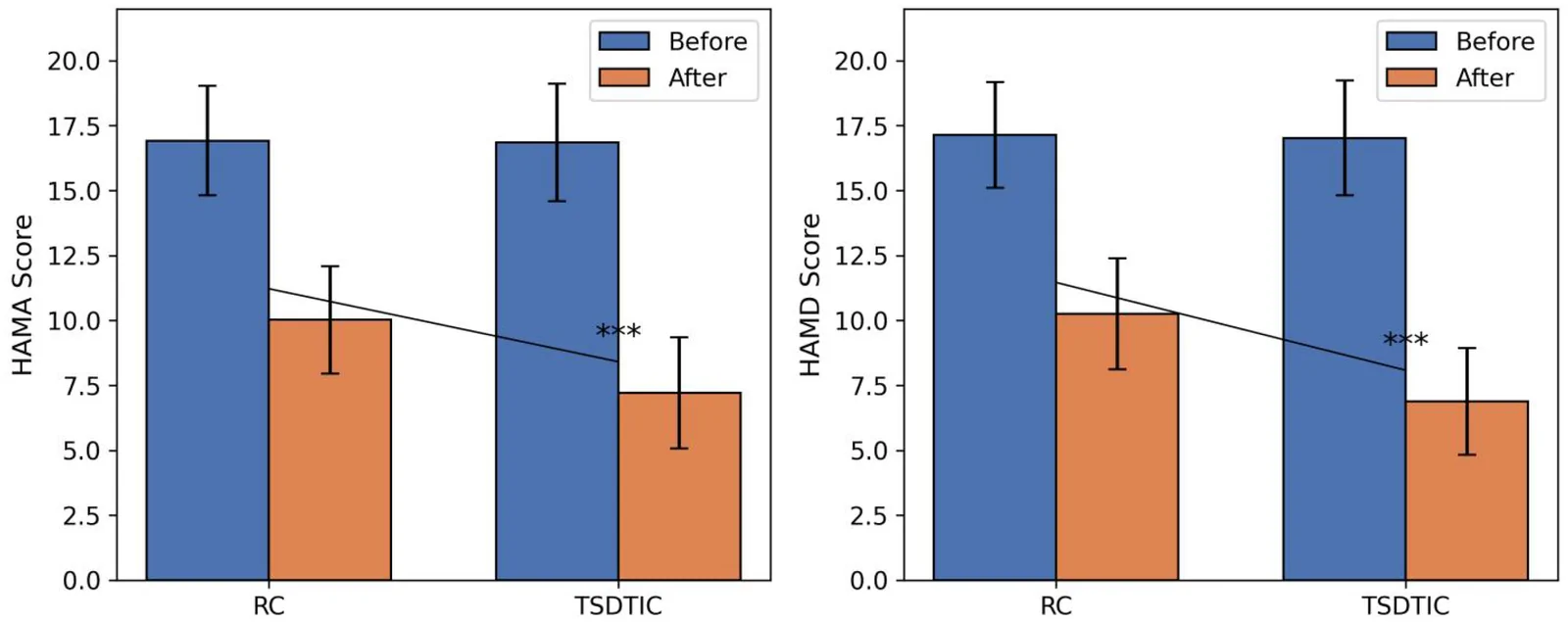
Comparison of Hamilton Anxiety Scale (HAMA) and Hamilton Depression Scale (HAMD) scores before and after nursing intervention between the two groups. **(A)** HAMA scores; **(B)** HAMD scores. Data are presented as mean ± standard deviation (SD). Before nursing: HAMA scores were 16.92 ± 2.10 in the RC group and 16.85 ± 2.26 in the TSDTIC group; HAMD scores were 17.14 ± 2.04 in the RC group and 17.02 ± 2.21 in the TSDTIC group (P>0.05 for all between-group comparisons, indicating comparable baseline). After nursing: HAMA scores decreased to 10.02 ± 2.06 in the RC group and 7.21 ± 2.13 in the TSDTIC group; HAMD scores decreased to 10.26 ± 2.14 in the RC group and 6.88 ± 2.06 in the TSDTIC group. The TSDTIC group showed significantly greater reductions in both HAMA and HAMD scores compared with the RC group. P<0.001 for all post-intervention between-group comparisons (independent-sample t-test). RC, routine care; TSDTIC, three-subject dual-track interactive care. ***P<0.001.

### 3.5 Quality of life

In TSDTIC group, physical function (70.35 ± 6.64 *vs*. 53.98 ± 5.36), role physical (68.68 ± 7.51 *vs.* 50.36 ± 7.53), body pain (65.14 ± 8.71 *vs*. 52.11 ± 8.08), vitality (69.92 ± 6.24 vs 55.12 ± 5.31), social function (66.08 ± 8.56 vs 51.80 ± 8.17), emotional function (67.77 ± 7.01 *vs*. 49.58 ± 7.62), mental health (72.21 ± 5.91 *vs*. 57.10 ± 4.72), and general health (68.91 ± 6.25 *vs*. 56.08 ± 4.91) were significantly higher than those in RC group (all P<0.001) (**Table 4**). After Bonferroni correction (α’=0.00625), all P values remained <0.001, confirming the robustness of these differences.

**Table 4:** The quality of life scores of the two groups were compared [(mean ± SD)].

Classification	RC group(n=84)	TSDTIC group(n=66)	t	P value
Physical function	53.98 ± 5.36	70.35 ± 6.64	16.70	<0.001
Role physical	50.36 ± 7.53	68.68 ± 7.51	14.81	<0.001
Bodily pain	52.11 ± 8.08	65.14 ± 8.71	9.47	<0.001
Vitality	55.12 ± 5.31	69.92 ± 6.24	15.69	<0.001
Social function	51.80 ± 8.17	66.08 ± 8.56	10.40	<0.001
Role emotional	49.58 ± 7.62	67.77 ± 7.01	15.03	<0.001
Mental health	57.10 ± 4.72	72.21 ± 5.91	17.43	<0.001
General health	56.08 ± 4.91	68.91 ± 6.25	14.08	<0.001

RC, routine care; TSDTIC, three-subject dual-track interactive care. After Bonferroni correction for 8 comparisons, the corrected significance level was α’=0.00625; all P values remained <0.001, confirming statistical significance.

### 3.6 Rate of satisfaction with nursing

Satisfaction with nursing in the RC group was distributed as follows: very satisfactory (*n* = 38); relatively satisfactory (*n* = 24); and unsatisfactory (*n* = 22). Satisfaction with nursing in the TSDTIC group was distributed as follows: very satisfactory (*n* = 35); relatively satisfactory (*n* = 28); and unsatisfactory (*n* = 3). The subjective satisfaction rate with nursing in the TSDTIC group was significantly higher than that in the RC group (95.45% *vs*. 73.81%; *P* < 0.001) (**Figure 2**). Given the exploratory nature of the self-designed questionnaire, this result should be interpreted with caution.

**Figure 2:**
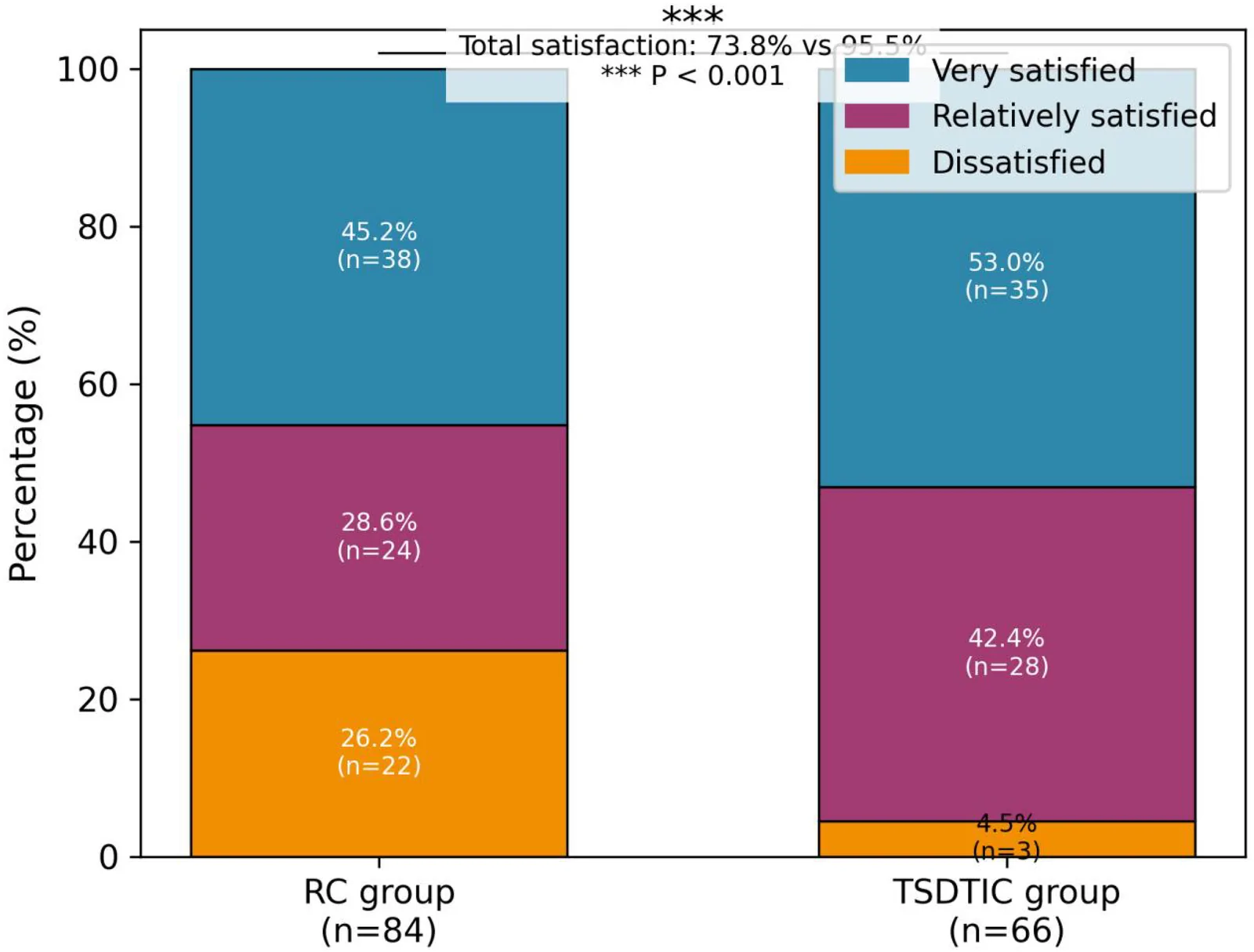
Comparison of nursing satisfaction between the RC group and the TSDTIC group. Satisfaction was categorized as very satisfied, relatively satisfied, or dissatisfied. Data are presented as percentages with raw counts in parentheses. The total satisfaction rate (very satisfied +relatively satisfied) was significantly higher in the TSDTIC group than in the RC group (95.45% vs. 73.81%; P<0.001). RC, routine care; TSDTIC, three-subject dual-track interactive care. The satisfaction questionnaire was self-designed and not formally validated; these results should be interpreted as exploratory.

## 4 Discussion

This retrospective cohort study systematically compared the effects of a three-subject dual-track interactive care (i.e., TSDTIC) model and routine nursing on wound healing, psychological state, and quality of life in hospitalized patients with DF. The results revealed that the effective rate of wound improvement, blood glucose control, psychological status score, quality of life, and satisfaction with nursing patients who received TSDTIC were significantly better than those of the RC group, indicating that the integrated nursing model was associated with better clinical outcomes for the comprehensive management of DF.

The effective rate of wound improvement in the TSDTIC group was significantly higher than that in the RC group, which may have been due to the systematic and multidisciplinary wound management strategies used in this model. Compared with RC, interactive nursing not only emphasizes standardized debridement and dressing selection but also integrates nutritional intervention and regular wound assessment and forms a closed-loop management led by specialist nurses. In addition, extended care outside the hospital ensured the continuous implementation of nursing measures through family follow-up and online support and reduced the delay of healing or recurrence of infection caused by nursing interruption after discharge from RC (24–26). Blood glucose stability is a core factor affecting DF healing. Persistent hyperglycemia can impair tissue repair and increase the risk for infection (27, 28). Fasting and postprandial blood glucose levels in the TSDTIC group after nursing were significantly lower than those in the RC group. This may be attributed to the precise blood glucose management and patient empowerment strategies implemented in this model. The nursing team established clear blood glucose control goals and dynamically adjusted them according to individual patient conditions. More importantly, through the one-on-one demonstration and feedback method, patients mastered the skills of self-monitoring and management of blood glucose, which improved their treatment compliance. However, treatment compliance and self-management skills were not directly measured as outcomes in this study; these proposed mechanisms remain hypothetical and warrant prospective investigation. This model potentially contributes to wound repair through a double-track linkage within and outside the hospital.

Anxiety and depression are common among patients with DF due to the risk for amputation, long-term treatment burden, and functional limitations. Negative emotions can further inhibit immune function and wound healing by activating the hypothalamic-pituitary-adrenal axis and increasing cortisol levels (29–31). Improvement in psychological status was an important finding in this study. Results revealed that HAM–A and HAM–D scores of the TSDTIC group were significantly lower than those of the RC group after nursing, which was closely associated with the inclusion of systematic psychological screening and professional counseling in this model. RC often lacks systematic psychological assessment and intervention. In the TSDTIC model, psychotherapists participate in a multidisciplinary team, conduct regular screening and counseling, and strengthen the social support network through family training. Through continuous interactions with medical staff and family members, patients gained more emotional support and a sense of disease control, thus alleviating helplessness and anxiety. Although the mediating effects of disease-control perception and social support on psychological outcomes were not directly quantified, these proposed mechanisms are consistent with established theoretical frameworks and warrant future validation. This suggests that the structured integration of psychological support into chronic wound care is important for improving overall patient outcomes. Quality of life is an important comprehensive index for evaluating the effects of chronic diseases in nursing (32). This study further confirmed the potential of a patient-centered, multi-agent, collaborative care model to improve overall patient health outcomes. The results revealed that the TSDTIC group had significantly higher scores than the RC group in all 8 dimensions of the SF-36, especially in terms of physical, mental, and social functioning. This indicates that this model not only focuses on the physiological level of wound healing but also comprehensively improves the functional status and life experience of patients by improving their self-management ability and enhancing their social support system. Patient satisfaction is not only an important reflection of the quality of care but is also closely related to treatment compliance and long-term prognosis (33). In this study, satisfaction with nursing in the TSDTIC group was significantly higher than that of the RC group, which may be due to enhanced nurse-patient communication, personalized and continuous nursing services provided by this model, and a greater sense of patient participation.

Regarding glycated hemoglobin (HbA1c), it is widely recognized as a critical marker of long-term glycemic control and microvascular risk in patients with diabetes (34, 35). The absence of HbA1c data in this study limits our ability to assess sustained glycemic management beyond the acute hospitalization period. HbA1c reflects average glycemic exposure over approximately 8–12 weeks and would provide valuable insight into whether the TSDTIC model produces durable metabolic benefits. Future prospective studies should incorporate HbA1c assessment at predefined intervals to evaluate the long-term metabolic impact of this integrated care model.

The strength of this study is that it evaluated the combined effects of a structured, multidimensional model of care in a real-world clinical setting using a retrospective cohort design. By combining professional medical support, patient self-management, and family and community social support, the three-subject, dual-track model ensures a seamless connection between in-hospital treatment and out-of-hospital rehabilitation, which is highly systematic and operable. The results of this study are highly consistent with the concept of integrated and continuous care for chronic diseases advocated in recent years.

### 4.1 Limitations

The present study had several limitations. First, the single-center, retrospective cohort design with historical control was used. This sequential historical-control design inherently confounds the intervention with calendar time; changes over time in wound-management protocols, glycemic management, staffing, antibiotic use, or hospital policies during the study period could contribute to observed differences. Although baseline characteristics were comparable, unmeasured time-varying factors could have influenced the outcomes. Second, the relatively small sample size and the lack of random allocation limit the generalizability and causal inference. Third, glycated hemoglobin (HbA1c) was not measured as an outcome indicator, which limits the assessment of long-term glycemic control; future studies should incorporate HbA1c to evaluate sustained glycemic management. Fourth, although WeChat group adherence and nurse-scheduled follow-up completion rates were monitored, the quality and depth of patient engagement in the WeChat group were not quantitatively assessed (only passive read-rates were used), and the reasons for missed follow-ups were based on retrospective chart review rather than prospective tracking. Additionally, the self-designed satisfaction questionnaire has not been validated, and the satisfaction results should be considered exploratory. Fifth, long-term outcomes, such as ulcer recurrence, rehospitalization, and amputation-free survival, were not tracked. Finally, we did not assess socioeconomic status or objective family support levels, which could affect the effectiveness of out-of-hospital care. In the future, it will be necessary to conduct multicenter, large-sample prospective randomized controlled trials and extend the follow-up to further verify the long-term and cost-effectiveness of this model.

## 5 Conclusion

The three-subject dual-track interactive care model was associated with significantly promote short-term wound healing, improve blood glucose control during hospitalization, relieve negative emotions, and improve quality of life and satisfaction with nursing of patients with DF. This model integrates medical staff, patients, and family members/communities to realize the integration of nursing within and outside the hospital, providing a new concept for the comprehensive management of DF. However, owing to the historical control design and lack of long-term follow-up, the sustained benefits require confirmation through prospective randomized studies. It is recommended to gradually promote this model in clinical practice and adjust it in combination with individualized assessment and resource conditions to optimize the overall nursing outcomes of patients with DF.

## Data Availability

The original contributions presented in the study are included in the article/supplementary material. Further inquiries can be directed to the corresponding author.
